# 2461. Carbapenem-resistant Enterobacterales reported to the National Healthcare Safety Network Multidrug-Resistant Organism & *Clostridioides difficile* Infection Module, January 2018 to July 2022

**DOI:** 10.1093/ofid/ofad500.2079

**Published:** 2023-11-27

**Authors:** Samuel E Cincotta, Rany Octaria, Walters Maroya

**Affiliations:** Centers for Disease Control and Prevention, Atlanta, Georgia; Centers for Disease Control and Prevention, Atlanta, Georgia; CDC, Atlanta, Georgia

## Abstract

**Background:**

To describe the COVID-19 pandemic’s impact on the spread of multidrug-resistant organisms, we compared pre-pandemic and pandemic laboratory-identified carbapenem-resistant Enterobacterales (CRE) events reported to the National Healthcare Safety Network (NHSN).

**Figure:**
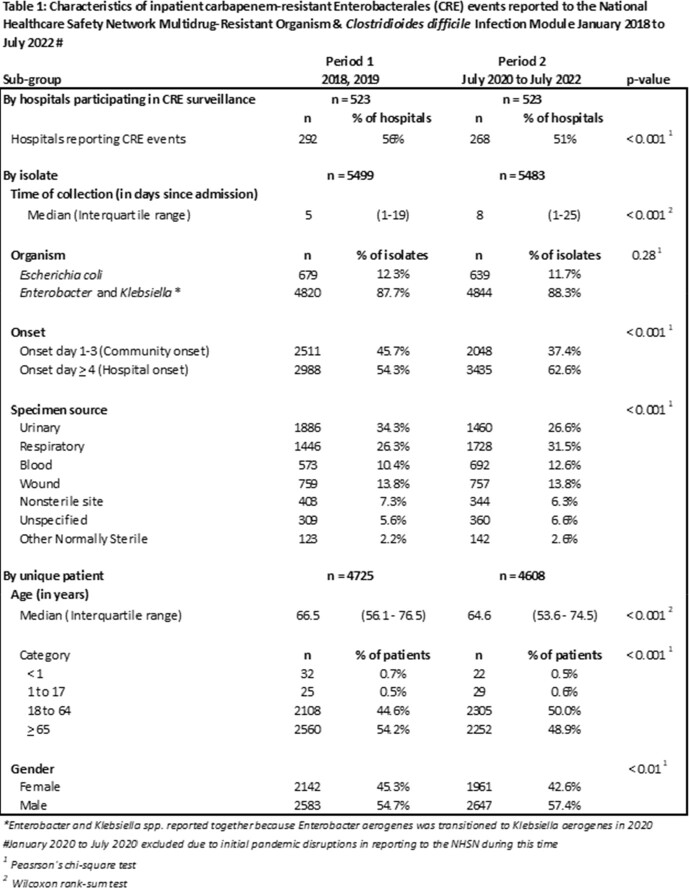

**Methods:**

A CRE event was a patient’s first *Escherichia coli*, *Klebsiella aerogenes*, *K. oxytoca*, *K. pneumoniae*, or *Enterobacter spp.* collected on an inpatient unit in a 14-day period and resistant to ≥ 1 of the following: imipenem, meropenem, doripenem, ertapenem, meropenem/vaborbactam (as of 2021), or imipenem/relebactam (as of 2021). We included events from acute care general hospitals (ACH) performing and continuously reporting facility-wide inpatient CRE surveillance for the same specimen sources in each month, from 2018 through 2019 (Period 1) and July 2020 to July 2022 (Period 2); the first half of 2020 was excluded due to initial pandemic disruptions in reporting to the NHSN. We compared patient demographics and epidemiological changes of events between the periods using Pearson’s χ^2^ or Wilcoxon rank-sum test as appropriate.

**Results:**

Overall, 523 ACHs in 28 states met inclusion criteria. During Period 1, there were 5499 events from 4725 patients in 22 states compared to 5483 events from 4690 patients in 22 states in Period 2; 21 states were consistent in both periods. More facilities had ≥ 1 event in Period 1 compared to Period 2 (56% versus 51%; p< 0.001). Four public health jurisdictions with mandates to report CRE to the NHSN accounted for 86.7% of Period 1 events and 86.0% of Period 2 events. Between Periods 1 and 2, the proportion of events that were hospital onset (on or after admission day four) (54.3% to 62.6%, p< 0.001), from respiratory sources (26.3% to 31.5%, p< 0.001), and bloodstream infections (10.4% to 12.6%, p< 0.001) increased, and the median patient age decreased (66.5 to 64.6, p< 0.001) (Table 1).

**Conclusion:**

Despite a consistent number of events in ACHs during the two periods, we observed a shift toward events that were hospital onset, associated with invasive infection, and occurring in younger people. The timely availability of robust laboratory-based surveillance is an important public health resource for understanding potential epidemiological changes in antimicrobial resistant threats.

**Disclosures:**

**All Authors**: No reported disclosures

